# Primary hepatic neuroendocrine tumor: A case report and literature review

**DOI:** 10.1016/j.ijscr.2020.05.057

**Published:** 2020-05-30

**Authors:** Adriano C. Costa, Fernando Santa-Cruz, Henrique Guimarães, Alexandre R. Paz, Eduardo A.C. Costa, José-Luiz Figueiredo, Álvaro A.B. Ferraz

**Affiliations:** a Oncological Surgery Unit, Napoleão Laureano Hospital, João Pessoa, PB, Brazil; b School of Medicine, Federal University of Pernambuco, Recife, PE, Brazil; c Department of Pathology, Napoleão Laureano Hospital, João Pessoa, PB, Brazil; d Department of Radiology, Napoleão Laureano Hospital, João Pessoa, PB, Brazil; e Department of Surgery, Federal University of Pernambuco, Recife, PE, Brazil

**Keywords:** Neuroendocrine tumors, Liver neoplasms, Immunohistochemistry, Diagnostic imaging

## Abstract

**Introduction:**

Primary hepatic neuroendocrine tumors (PHNETs) are extremely rare, having less than 200 cases reported in the literature.

**Presentation of case:**

A 51-year-old woman presenting with mild intensity sharp pain in the upper abdomen that started 4 years ago. Preoperative Magnetic Resonance Imaging (MRI) suggested hepatocellular adenoma. Surgical exploration found a tumor occupying the whole segment IV of the liver, therefore a left hepatectomy was performed. Pathology and immunohistochemistry indicated to be a low-grade neuroendocrine tumor of the liver. The patient is enjoying a good quality of life, free of disease, presenting no signs of recurrence nor metastases 12 months after the procedure.

**Conclusion:**

The clinical presentation of PHNETs is non-specific and its radiologic features are intensely diversified, frequently leading to misdiagnosis of other hepatic neoplasms.

## Highlights

• Primary hepatic neuroendocrine tumors are rare neoplasms, with less than 200 cases reported.
• The clinical presentation of PHNETs is non-specific and its radiologic features are intensely diversified.
• Surgical resection with clear margins is the mainstay treatment for PHNETs, with very positive results.
• For unresectable tumors, liver transplantation and transcatheter arterial chemoembolization are the main options.

## 1 Introduction

Neuroendocrine tumors (NETs) are a type of low malignancy tumors that arise in the neuroendocrine cells throughout the body, originated from primary cells of the neural crest that rarely migrate to the liver. It is originated more often from the gastrointestinal (GI) tract, lungs, pancreas, gallbladder, thymus, ovaries and testes [[1], [2], [3]]. PHNETs can only be clinically diagnosed after excluding the possibility of extrahepatic disease with metastasis to the liver [4]. However, due to its non-specific presentation, the diagnosis of these hepatic neoplasms before pathologic evaluation of a surgically resected specimen is very challenging [3]. It was first described by Edmondson et al. in 1958, [7] since then, less than 150 cases of PHNETs were described in the literature [8]

Herein, we report a case of PHNET, approached through surgical resection, and diagnosed postoperatively by immunohistochemistry (IHC). This work has been reported in line with the SCARE criteria [9].

## 2 Case report

**Fig. 1:**
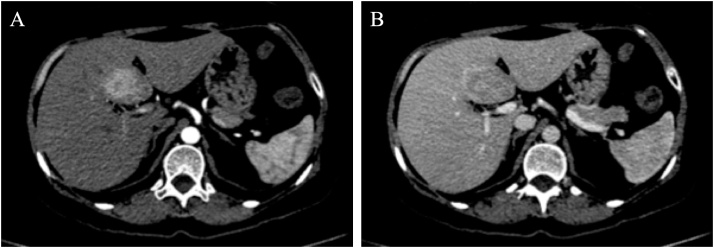
Preoperative CT scans: arterial phase **(A)**; and portal phase **(B)**.

A 51-year-old woman was referred to the Surgical Oncology Unit of the Napoleão Laureano Hospital, João Pessoa, PB, Brazil, presenting with a history of mild intensity sharp pain in the upper abdomen that started 4 years ago, and have worsened in the last 6 months before the consultation. The patient did not present any comorbidities and denied medication usage. At physical examination, there was no palpable mass, but a mild abdominal tenderness, especially in the upper quadrants, was evidenced. There were no signs of jaundice nor alterations in others systems. Laboratory tests indicated the following measures: Hemoglobin 10.1 g/dL, AST 134.0 U/mL, ALT 203 U/mL, prothrombin activity 39.8 %, prothrombin time 20.8 s, INR 1.6. Gamma-glutamyl transpeptidase (GGT), serum alkaline phosphatase (ALP), bilirubin, amylase and albumin were within their normal range. The patient has previously performed a computed tomography (CT) scanning in other Unit, which showed a tumor of 5 cm in the segment IV of the liver, with contrast-enhancement in the arterial phase (Fig. 1). Chest X-ray did not show any alterations. A Magnetic Resonance Imaging (MRI) with liver-specific contrast agent, which evidenced a liver containing a subcapsular oval lesion with lobulated contours, measuring 4.3 × 2.8 cm, with T1-hypointense signal and T2-hyperintense signal, located within the segment IV-B (Fig. 1). It has shown high contrast-enhancement in the arterial phase (Fig. 1-A), with washout and without capsule, aside with hypointense contrast-enhancement in the hepatobiliary phase (Fig. 1-C). The MRI report suggested hepatocellular adenoma, therefore, preoperative percutaneous liver biopsy was not indicated.

**Fig. 2:**
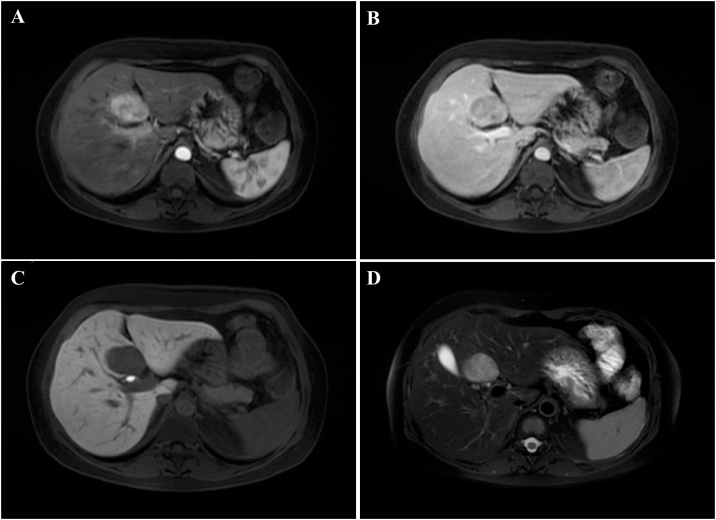
Preoperative MRI scans: T1-weighted image in the arterial phase **(A)**; T1-weighted image in the portal venous phase **(B)**; T1-weighted image in the hepatobiliary phase **(C);** pre-contrast T2-weighted image with fat-sat.

Based on these findings, on July 6th 2018, a laparotomy was performed. Surgical exploration found a tumor occupying the whole segment IV of the liver. The tumor was resected by left hepatectomy (segments IV, II and III) using cavitron ultrasonic surgical aspirator (CUSA), associated with cholecystectomy (Fig. 2).

The postoperative course was uneventful and the patient was discharged at the fifth postoperative day.

**Fig. 3:**
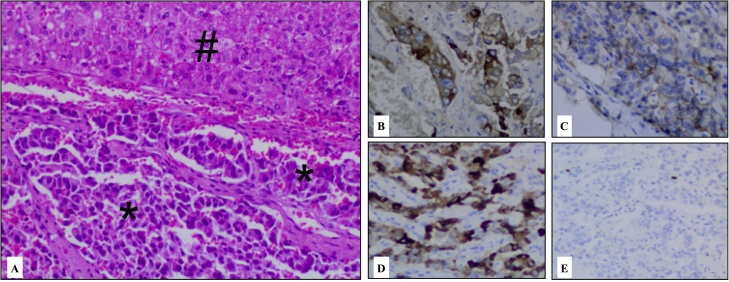
Neuroendocrine Tumor (*) infiltrating the hepatic parenchyma **(#)** stained with Hematoxylin and Eosin (H&E) – 200x **(A**); positive IHC staining for Chromogranin A **(B)**, CD56 **(C)**, CK-7 **(D)** and low Ki-67 proliferation index **(E)**.

Pathology suggested and immunohistochemistry (IHC) confirmed to be a low-grade neuroendocrine tumor of the hepatic parenchyma (Fig. 2). There were 4 mitotic figures per 10 high power fields. The IHC staining were positive for CK7 (SP52), chromogranin A (LK2H10) and CD56 (123C3), and Ki-67 were lower than 5%. Other immunohistochemical staining were studied, such as CK20 (SP33), glypican3 (GC33), GATA3 (L50-823) and TTF-1 (8G7G3/1), and all of them were negative (Fig. 3).

Due to the preoperative diagnosis of hepatocellular adenoma, octreotide scintigraphy (OctreoScan) was not indicated to stage the tumor. However, in the postoperative period, after the confirmation of NET by the IHC, the patient was submitted to OctreoScan, and no tumor sites were found.

Currently, 12 months after the surgical procedure, the patient is enjoying a good quality of life, free of disease, presenting no signs of recurrence nor metastases.

## 3 Discussion

PHNETs usually present silent manifestations, without endocrinological repercussions rarely leading to hormone-related symptoms, contrasting with hepatic metastases from other organs NETs [3]. Its incidence appears to be similar between men and women, however some studies have found a higher number of males affected within their sample. The mean age of onset is 47 years old [1]. These tumors have a slow growth and only become clinically evident at advanced stages. Clinical manifestations include weight loss, fatigue, abdominal distension, pain and palpable mass in the right upper quadrant [3,4].

Preoperative imaging frequently misdiagnoses PHNETs, since these lesions exhibit miscellaneous radiological features. Although inconclusive, the PET/CT scans usually show low density on the lesions site and a contrast-enhancement in the arterial phase [10,11]. Classic tumor markers, such as AFP, CEA and CA19-9 are usually negative [3,4]. Furthermore, there is still no consensus among authors whether preoperative liver biopsy is an efficient diagnostic tool for these neoplasms, and so, postoperative anatomopathological and immunohistochemical evaluations stand as the mainstay for the definitive diagnosis [3].

PHNETs most commonly present slow growth and low malignancy potential. The assessment of cellular proliferation appears as a reliable tool to determine the malignancy potential of these tumors, and lower cellular proliferation is linked to better survival rates [4]. The current 2019 WHO classification includes three grades (G1, G2 and G3) for NETs, which are related to the mitotic index and ki-67 index: NET G1 presents, respectively <2 and < 3 %, while NET G2 is 2–20 and 3–20 %, and NET G3 > 20 and >20 %, respectively [12]. The current case presented a low cellular proliferation index, as evidenced by the low Ki-67, and 4 mitotic figures per 10 high power fields, namely it is expected a better prognosis for this case, graded as a G2 according to the WHO. Furthermore, it is not well established if the tumor number presents relationship with the prognosis or not, having studies that found positive correlation and others that refuted [4,5]. It is important to keep a long-term follow-up with periodic imaging tests due to the need of excluding an eventual primary site of the disease different from that of first presentation [13].

Generally, these tumors are related to good survival rates, presenting a 10-year survival as high as 73 % [6]. Contrastingly, a series of 22 cases of PHNETs, showed a recurrence rate of 40.9 % and a 5-year overall survival rate of 64.7 % [4]. The present case does not have a long-term follow-up evaluation, however, currently, 12 months after the surgical procedure, the patient is alive, free of disease, presenting no signs of recurrence or metastasis.

Surgical resection with clear margins is the mainstay treatment for PHNETs, since there is a resectability rate of 70 % of the tumours, and surgery have shown very attractive results regarding the long-term survival rates [6,14]. For those even rarer cases, where the tumor is unresectable, there are other treatment options, including liver transplantation and transcatheter arterial chemoembolization (TACE) [6]. TACE associated to systemic chemotherapy is the main option for these cases of unresectable tumors, however the outcomes are poor [4]. In the current case, the patient was treated through the resection of segments IV, II and III of the liver, associated with cholecystectomy.

## 4 Conclusion

PHNET is an extremely rare tumor that exhibits slow growth and low malignancy potential. Its clinical presentation is non-specific, and its radiologic features are intensely diversified, frequently leading to misdiagnosis of other hepatic neoplasms. Furthermore, surgical resection with clear margins is still the best therapeutic modality in terms of improving survival rates.

## Declaration of Competing Interest

The authors declare no potential conflict of interest related to this manuscript.

## Sources of funding

This research did not receive any specific grant from funding agencies in the public, commercial, or not-for-profit sectors.

## Ethical approval

This study was approved by the Ethics Committee of Federal University of Pernambuco, under the registration number CAAE 28005220.8.0000.5208.

## Consent

Written informed consent was obtained from the patient for publication of this case report and accompanying images. A copy of the written consent is available for review by the Editor-in-Chief of this journal on request.

## Author’s contribution

Study concept and design: AABF, ACC.

Data Collection: FSC, HG, EACC, ARP.

Data Analysis and interpretation: HG, JLF, ARP, EACC.

Writing the paper: FSC, ACC.

Revision: JLF, AABF.

## Registration of research studies

This study was approved by the Ethics Committee of Federal University of Pernambuco, under the registration number CAAE 28005220.8.0000.5208.

## Guarantor

Adriano Carneiro da Costa (adrianocacosta@gmail.com).

## Provenance and peer review

Not commissioned, externally peer-reviewed.
